# RIBDB: An SRS Based Infrastructure for REALIS

**DOI:** 10.1002/cfg.139

**Published:** 2002-02

**Authors:** Peter Rice, Bijay Jassal, Antoine de Daruvar

**Affiliations:** 1 LION Bioscience Ltd Compass House 80-82 Newmarket Rd Cambridge CB5 8DZ UK; 2 LION bioscience AG Waldhofer Str. 98 Heidelberg D-69123 Germany

## Abstract

The REALIS project is an EU-funded consortium for the post genomic analysis of the food pathogen *Listeria monocytogenes.* The data generated by the consortium members is stored under the RIBDB database, a system built using SRS which integrates consortium data, public databases, and applications for analysis. RIBDB is available to all consortium members through a web server, with the option of installing a local mirror of the main server for local analysis.


**Comparative and Functional Genomics**
*Comp Funct Genom* 2002; **3**: 35–36.
**DOI:** 10.1002/cfg.139

**Conference Review**

### RIBDB: An SRS based infrastructure for REALIS

Peter Rice[1]*, Bijay Jassal[1] and Antoine de Daruvar[2]
[1] *LION Bioscience Ltd, Compass House, 80-82 Newmarket Rd, Cambridge, CB5 8DZ, UK*
[2] *LION bioscience AG, Waldhofer Str. 98, D-69123 Heidelberg,Germany*

*Correspondence to:
*LION Bioscience Ltd, Compass House, 80-82 Newmarket Rd, Cambridge, CB5 8DZ, UK.
E-mail:
peter.rice@uk.lionbioscience.com*

Received: 6 December 2001
Accepted: 6 December 2001
Published online:
9 January 2002

### Abstract

**The REALIS project is an EU-funded consortium for the post genomic analysis of the food pathogen *Listeria monocytogenes*. The data generated by the consortium members is stored under the RIBDB database, a system built using SRS which integrates consortium data, public databases, and applications for analysis. RIBDB is available to all consortium members through a web server, with the option of installing a local mirror of the main server for local analysis. Copyright © 2002 John Wiley & Sons, Ltd.**

**Keywords: bioinformatics; SRS; REALIS; Listeria**

### SRS overview

SRS was originally developed [2] as an indexing and querying system for databanks in text or flatfile formats. It is now a powerful data integration platform, providing rapid, easy and user friendly access to the large volumes of diverse and heterogeneous Life Science data. SRS indices provide rapid query capabilities on large volumes of text data. The databanks are linked in a network for rapid navigation and sophisticated querying across databases. SRS is available free of charge to academic users, and over 400 databases are maintained on more than 100 public SRS servers. Recent additions to SRS include the capability to directly use data in XML format and in relational databases.

Integration is achieved partly by standardising fields which are equivalent between databases, for example converting all dates and all author names into a single standard format for querying and cross referencing. By providing a standard view of a large set of databases, SRS allows users to query all the databases for any common field. In this way, for example, all the sequence and structure databases can be searched for an author name or date without the need to know the internal author or date format. Complex queries across many databases can be combined, for example author and description, or 'all text' which is a simple 'quicksearch' option on the top page.

Databases are linked by using shared common fields (for example SwissProt accession numbers in protein cross reference fields), or by locating identifiers in the text (for example Enzyme Commission numbers in functional annotation). Once databases have been linked, queries can navigate across databases to rapidly find, for example, all proteins sharing a common motif as defined in their cross references to any of the motif databases.

Sophisticated queries can be performed in multiple steps through the web interface. Where these are in frequent use, they can be combined as 'Canned Queries' and made available from a menu.

The results of queries can be presented as plain text, but this is not friendly for most users. SRS usually parses the results, with the same code as it used for indexing, to extract entry ID, description, author, features, and other common fields. These are built into internal 'objects' which can then be used by 'views'. In this way, a plain sequence database entry, for example from EMBL or SwissProt, can be presented to the user in a common and readable form, with the possibility of using graphical views to display (for example) the sequence features.

Copyright © 2002 John Wiley & Sons, Ltd.

The internal 'object' built from one entry can be made more complicated by including data from other databases. For example, a view of a SwissProt entry can include data extracted from linked Prosite motifs, extracted directly from the Prosite database.

Application programs can be launched within SRS. Several public servers have implemented homology search programs such as BLAST and FASTA, and other analysis applications such as CLUSTALW.

### RIBDB (REALIS Integrated Bioinformatics Database)

The primary goal of RIBDB is to integrate all the data generated by the consortium members in a single server, and to provide support for specialized querying and reporting across the various REALIS work packages.

The initial RIBDB server was populated with the major public biological databases (EMBL, Swiss Prot, SpTrEmbl, Prosite, Blocks, Prints, Pfam, InterPro, Enzyme, Taxonomy, PDB, and KEGG pathways [1]). These databases are installed using the standard public SRS definitions and parsers.

Consortium data installed to date includes genome annotation from *Listeria monocytogenes* and *Listeria innocua* [3], and proteomics data from 2D gels generated by GBF in Braunschweig [4]. This is supplemented by additional databases, for example annotation of *Bacillus subtilis*, provided by consortium members and linked to these and to the public databases. The integration of these databases into SRS uses parsers custom developed for RIBDB to identify fields of special interest to the consortium members and to enforce standardisation of gene naming (for example) across all data sources so that queries can navigate across databases to use linked information.

Returning the original text data is accepted by most bioinformatics specialists, but is not convenient for most laboratory biologists. RIBDB includes custom views of the underlying source data to translate the original text format into one or more report formats. Examples of this can be seen for SwissProt on the EBI public server [1].

RIBDB includes the standard set of applications supported by SRS (BLAST, FASTA, HMMER, CLUSTALW, and others) which users can launch to analyze any sequence data retrieved by their queries.

### Planned extensions

We naturally anticipate more data from the other REALIS work packages in the very near future. Customized parsers and views for each of these data formats are designed and waiting for the availability of the first data sets.

REALIS users have begun to use the RIBDB server, and the feedback on the most common queries will be used to create custom 'Canned Queries' to simplify their future use of the server.

As the amount and diversity of sequence data increases, we are adding sequence analysis applications to the server so that querying can extend beyond the data provided in the source databases and include users' own analysis results. SRS launches applications by defining an interface for users to select run time options, launching the application, and parsing the result file in the same way as it parses a biological database. In this way, the application results become part of the user's private view of the RIBDB data.

### References

1. EBI SRS Server **http://srs.ebi.ac.uk/** (November 2001).
2. Etzold T, Argos P. 1993. SRS an indexing and retrieval tool for flat file data libraries. *Comput Appl Biosci* **9**: 49–57.
3. Glaser P, Frangeul L, Buchrieser C. 2001. Comparative genomics of *Listeria* species. *Science* **294**: 849–852.
4. Kaerst U. and the REALIS consortium 2002. REALIS: Postgenomic analysis of *Listeria monocytogenes*. *Comp Funct Genom* **3**(1).

Copyright © 2002 John Wiley & Sons, Ltd.
